# Letter from Chicago

**Published:** 1905-05

**Authors:** George H. Simmons

**Affiliations:** Editor Journal American Medical Association


### Letter from Chicago.

THE EDITOR OF THE JOURNAL OF THE AMERICAN MEDICAL ASSOCIATION ANNOUNCES THE COUNCIL ON PHARMACY AND CHEMISTRY.

*Editor Buffalo Medical Journal:*

SIR—I enclose a copy of the council on pharmacy and chemistry, a body created by resolution of the board of trustees of the American Medical Association at its last meeting. Technically, this is an advisory committee on ethical preparations as they relate to the advertising pages of the *Journal of the American Medical Association;* as a matter of fact, however, the functions of this council are more than this, for it will pass on preparations whether offered for advertisement or not, and those which are approved will be incorporated in the book, "New and Non-Official Remedies."

The need of a book of this character cannot be questioned. It is demanded in the interest of honest pharmacy and chemistry, but, above all, by physicians who need a work to which to refer for information regarding the character, reliability, etc., of the various preparations mentioned in medical literature, and brought to their attention in various ways, as well as to assist them in separating the secret nostrums from preparations which are honestly made and ethically exploited, and which are worthy of their recognition.

Thus there will be provided a consistent and equitable standard to which articles must conform to be acceptable for the advertising pages of the *Journal of the American Medical Association,* and at the same time a book of reference that will be of incalculable value to physicians. The same standard will also govern the mention of articles in the reading pages.

The members of the council are: Arthur R. Cushny, Ann Arbor; C. Lewis Diehl, Louisville; C. S. N. Hallberg, Chicago; Robert A. Hatcher, New York; L. F. Kebler, Washington; J. H. Long, Chicago; F. G. Novy, Ann Arbor; W. A. Puckner, Chicago; Samuel P. Sadtler, Philadelphia; J. O. Schlotterbeck, Ann Arbor; George H. Simmons, Chicago; Torald Sollmann, Cleveland; Julius Stieglitz, Chicago; M. I. Wilbert, Philadelphia; H. W. Wiley, Washington.

I am also enclosing a galley proof of an editorial which, with the announcement, will appear in the *Journal* next Saturday, March 4. We shall be glad to have you reproduce any or all of this, as you may desire. We hope the movement will receive your endorsement and support. Criticisms and suggestions are asked for.

GEORGE H. SIMMONS,
Editor *Journal American Medical Association.*

